# A Duplex Fluorescent Microsphere Immunoassay for Detection of Bluetongue and Epizootic Hemorrhagic Disease Virus Antibodies in Cattle Sera

**DOI:** 10.3390/v13040682

**Published:** 2021-04-15

**Authors:** Barbara S. Drolet, Lindsey M. Reister-Hendricks

**Affiliations:** Center for Grain and Animal Health, Agricultural Research Service, Arthropod-Borne Animal Diseases Research Unit, United States Department of Agriculture, Manhattan, KS 66502, USA; lindsey.reister-hendricks@usda.gov

**Keywords:** fluorescent microsphere immunoassay, FMIA, bluetongue virus, epizootic hemorrhagic disease virus, cattle, antibody, serology

## Abstract

Bluetongue virus (BTV) causes internationally reportable hemorrhagic disease in cattle, sheep, and white-tailed deer. The closely related, and often co-circulating, epizootic hemorrhagic disease virus causes a clinically similar devastating disease in white-tailed deer, with increasing levels of disease in cattle in the past 10 years. Transmitted by *Culicoides* biting midges, together, they constitute constant disease threats to the livelihood of livestock owners. In cattle, serious economic impacts result from decreased animal production, but most significantly from trade regulations. For effective disease surveillance and accurate trade regulation implementation, rapid, sensitive assays that can detect exposure of cattle to BTV and/or EHDV are needed. We describe the development and validation of a duplex fluorescent microsphere immunoassay (FMIA) to simultaneously detect and differentiate antibodies to BTV and EHDV in a single bovine serum sample. Performance of the duplex FMIA for detection and differentiation of BTV and EHDV serogroup antibodies was comparable, with higher sensitivity than commercially available single-plex competitive enzyme-linked immunosorbent assays (cELISA) for detection of each virus antibody separately. The FMIA adds to the currently available diagnostic tools for hemorrhagic orbiviral diseases in cattle as a sensitive, specific assay, with the benefits of serogroup differentiation in a single serum sample, and multiplexing flexibility in a high-throughput platform.

## 1. Introduction

Bluetongue virus (BTV) and epizootic hemorrhagic disease virus (EHDV) are midge-transmitted orbiviruses (Reoviridae) that cause devastating, re-emerging hemorrhagic diseases in livestock and wildlife. Of more than 30 BTV serotypes worldwide, BTV-2, -10, -11, -13, and -17 are considered endemic to the U.S. Of the seven EHDV serotypes, EHDV-1, -2, and -6 are considered endemic. These transboundary diseases are of particular concern to the cattle industry because of the emergence of new serotypes with unknown virulence [1,2,3,4], increased reports of clinical disease [5,6,7,8,9] and transplacental transmission [10,11,12,13,14,15,16,17] in cattle, and increased spread and adaptation of vectors and viruses to new geographic areas [18]. In the U.S., losses to BTV are conservatively estimated at $144 million annually, attributed to effects on animal health, production, and reproduction, but most significantly due to non-tariff trade restrictions on the sale and movement of animals and animal products [19,20,21]. With its considerable animal health and economic impact, bluetongue disease is a World Organization for Animal Health (OIE)-reportable disease [19,22]. Control methods for BTV include limited vaccines [23,24,25,26], livestock management [27,28], and vector control [29], with EHDV control often emulating standards set by BTV research [30,31,32].

Infections of cattle with BTV and EHDV are often subclinical. Domestic and international trade barriers imposed on the cattle industry are based on prolonged viremias, with cattle serving as amplifying reservoirs for biting midge transmission [33,34,35,36]. However, clinical disease has been reported in cattle for both BTV [6,7,8,10,11,12,13,14,15,16,17] and EHDV [37,38,39,40,41], and can include weight loss, reduced milk yields, lameness, fever, dehydration, still-births, and abortions. A presumptive diagnosis may be indicated by clinical signs; however, diagnostic tests are necessary for accurate diagnosis and trade regulation.

Antibody response in cattle typically develops 7–14 days post-infection (dpi) [42] and can be lifelong [41,43,44]. The most routine serological tests performed by the National Animal Health Laboratory Network across the U.S. include agar gel immunodiffusion (AGID) and competitive enzyme-linked immunosorbent assays (cELISA) [43,44,45]. Neither assay can simultaneously detect and differentiate antibodies to both orbiviruses in the same serum sample. Compared to the cELISA, the AGID test is less technical and more economical, but has lower sensitivity, lower specificity due to cross-reactivity between the two orbiviruses, and it is not high throughput [46,47,48]. While antibody cross-reactivity within BTV and EHDV serogroups is important for the ability of any serological assay to determine orbivirus exposure in cattle, regardless of the specific serotype with which they were exposed, cross-reactivity between the closely related viruses results in false positives which can have devastating, erroneous impacts on trade and compromises geographic disease risk modeling. Thus, the OIE states that AGID results are not appropriate for declaration of an individual animal being free from infection prior to movement, nor for confirmation of clinical cases [49].

Fluorescent microsphere immunoassays (FMIA) are relatively new diagnostic tools for rapid, sensitive, specific detection of multiple analytes in a single sample in a high-throughput platform. Using Luminex xMAP technology, with the MAGPIX^®^ system, incorporates magnetic microspheres in a lower cost, smaller footprint model than available with the first-generation model, and is more accessible for use by researchers and veterinary diagnostic laboratories. We developed a duplex microsphere assay based on Luminex xMAP technology to simultaneously detect and differentiate antibodies to BTV and EHDV in a single cattle serum sample. Both sensitivity and specificity of the microsphere assay were compared with commercially available BTV and EHDV cELISA kits.

## 2. Materials and Methods

### 2.1. Cells and Viruses

Baby hamster kidney cells (BHK; ATCC, Manassas, VA, USA) were grown in 490 cm^2^ roller bottles (Corning Costar, Corning, NY, USA) with Eagles MEM with Earl’s salts media (Sigma Aldrich, St. Louis, MO, USA) containing 1× antibiotic-antimycotic (Gibco, Gaithersburg, MD, USA) and 2% FBS (Atlanta Biologicals, Flowery Branch, GA, USA) at 37 °C with 5% CO_2_. For whole-virus antigen preparation, monolayers (80% confluence) were inoculated with BTV-17 or EHDV-2 at 0.1 MOI and incubated at 5% CO_2_ for 5–7 days until monolayers displayed 80–90% cytopathic effect. Sloughed cells were freeze–thawed twice at −80 °C and centrifuged at 1800× *g* for 10 min at 4 °C to remove cellular debris. Virus was pelleted from cleared supernatant through a 25% sucrose cushion by ultracentrifugation (Beckman Coulter, Indianapolis, IN, USA) at 28,000× *g* for 1 h at 4 °C. Virus pellets were resuspended in 1 mL of sterile phosphate buffered saline (PBS, pH 7.2) and filtered through a 0.45 µm syringe filter (Millipore, Burlington, MA, USA). An aliquot of the purified virus was titered by standard plaque assay on Vero MARU cells (VM; Middle America Research Unit, Panama) grown in 199E media at 37 °C with 5% CO_2_. To the remaining purified virus, Tween 20 (Fisher Scientific, Hampton, NH, USA) was added at a final concentration of 0.1% and the tube vortexed for 10 s. Protein concentrations of whole-virus antigen preparations were measured on a Nanodrop spectrophotometer (Fisher Scientific) and stored at 4 °C for up to six months.

### 2.2. Microsphere Conjugation

Whole-virus antigen was coupled to carboxylated Luminex MagPlex^®^ polystyrene magnetic microsphere beads (Luminex Corporation, Austin, TX, USA) using the basic two-step carbodiimide reaction, according to the manufacturer’s instructions, utilizing regions #20 and #56. Briefly, 1.24 × 10^6^ beads in 1 mL of storage buffer were placed in an opaque 1.7 mL tube and placed on a magnetic separator for 2 min. Storage buffer was carefully removed, beads were resuspended in 1 mL deionized water, vortexed for 20 s, and sonicated (Qsonica, Newtown, CT, USA) at 100 mV for 20 s, constant. Washed beads were again placed on the magnet, deionized water was removed, and beads were activated with 80 µL 0.1 M sodium phosphate, pH 6.2. After vortexing for 20 s, 20 µL of N-hydroxysulfosuccinimide (Sulfo-NHS) (Pierce Protein, Waltham, MA, USA) at 50 mg/mL was added and tube was vortexed briefly to mix. Next, 10 µL of freshly made 50 mg/mL EDC cross-linker (Thermo Scientific) was added, the tube vortexed briefly to mix, and incubated at room temperature for 20 min, vortexing for 10 s every 10 min. Activated beads were then separated from the liquid as above by magnet and washed twice with 500 µL coupling buffer; 50 mM MES (Sigma Aldrich, St. Louis, MO, USA), pH 5.0 at 4 °C. Whole-virus BTV antigen or EHDV antigen in PBS was added to #20 and #56 microbeads, respectively, at 10 µg/1 million beads. Beads were vortexed for 20 s and placed on a tube rotator for 2 h at room temperature in an opaque 1.7 mL tube. The pellet was washed twice as above with 1 mL of PBS-TFN and resuspended in 250 µL of PBS-TFN. Conjugated bead sets were counted on a Bright Line hemacytometer (Hausser Scientific, Horsham, PA, USA) and stored in an opaque 1.7 mL tube at 4 °C for up to 1 year.

### 2.3. Cattle Sera

Commercially available BTV/EHDV-negative sera were evaluated against known negative pre-bleed sera for use as a negative control. Sera included unfiltered normal cattle sera (Lampire Biological Laboratories, Pipersville, PA, USA), filtered normal cattle sera (Sigma Aldrich, St. Louis, MO, USA), normal cow sera (GeneTex, Irvine, CA, USA), and negative control sera from BTV and EHDV cELISA kits (Veterinary Diagnostic Technology, Inc, Colorado). Positive controls included sera from cattle experimentally infected with BTV-10, -11, -13, or -17, or with EHDV-1 or -2, at 42–49 dpi with positive AGID results. All controls were tested in triplicate and average mean fluorescence intensity (MFI) values were directly compared to previous cELISA or AGID data. Negative and positive control sera were tested at 1:100, 1:250, and 1:500 in phosphate buffered saline, pH 7.4, with 0.05% Tween-20 (PBST) to determine optimal dilution for the assay. Field-collected test sera were run blind and included 202 archived cattle sera collected in Wyoming and Colorado for surveillance from June to August of 1984 (*n* = 113) and collected in South Dakota and Nebraska from October to December following the 2012 EHD outbreak [50] (*n* = 89). All sera had previously been tested for BTV and EHDV antibody either by AGID or cELISA.

### 2.4. Fluorescent Microsphere Immunoassay (FMIA)

For the assays, approximately 500 whole-virus antigen-conjugated beads per virus, suspended in 100 µL PBS-TFN, were placed in each well of an opaque 96-well round bottom plate (Corning Costar). Serum samples were diluted 1:100 in PBST, 100 µL were added to each well, the plate was sealed with Axygen foil sealing tape (Corning) and incubated at room temperature with gentle shaking for 1 h. Beads were then washed three times using a Bio-Tek ELx405 (Bio-Tek, Winooski, VT, USA) 96-well magnetic plate washer. Briefly, plates were placed on the magnet for 3 min, foil tape was removed, liquid was aspirated, 100 µL of PBST was delivered into each well, shaken for 1 min, placed on the magnet for 3 min, and liquid aspirated. Rabbit anti-bovine IgG secondary antibody conjugated with biotin (Abcam 13767, Abcam Cambridge, UK) was added to each well (100 µL) at a dilution of 1:2500 in PBST. Plates were sealed and incubated at room temperature with gentle shaking for 45 min, then washed twice as above. Next, 100 µL of streptavidin conjugated to R-Phycoerythrin (SAPE) (Invitrogen, Hampton, NH, USA) was added to each well at a dilution of 1:200 (0.05 mg/mL) in PBST. Plates were covered and incubated at room temperature with gentle shaking for 30 min. Plates were washed twice as above, beads were resuspended in 100 µL PBST and analyzed on a Bio-Plex MagPix (Bio-Rad, Hercules, CA, USA and Luminex Corporation, Austin, TX, USA) as per manufacturer’s instructions. A minimum of 100 microbeads per set were read and an MFI value was obtained for each bead set per well.

Results were collected with Bio-Rad MP Manager software (Bio-Rad) and initially analyzed with Bio-Plex Manager 6.1 software (Bio-Rad). GraphPad Prism software version 9.1.0 (GraphPad Software, Inc., La Jolla, CA, USA) was used to analyze single-analyte and duplex data via an ordinary one-way ANOVA with a Dunnett’s multiple comparisons test (*p* < 0.05) to determine statistical differences. To allow the comparison of data acquired between plates to known previous serological data and statistical analysis via receiver operating characteristic (ROC), the average MFI values from each target microbead set per sample were converted to sample value/positive value (S/P) ratios utilizing the following calculation:

$$\frac{S}{P}=\frac{sample\;MFI-negative\;control\;MFI}{positive\;control\;MFI-negative\;control\;MFI}$$

MedCalc statistical software version 19.7.4 (MedCalc Software, Ltd., Ostend, Belgium) was used to determine specificity and sensitivity via ROC analysis followed by a Youden’s Index (J statistic) to rate the performance of the assay and inform cutoff values within the sera tested.

### 2.5. cELISA

As a diagnostic comparison, commercially available, virus-specific, cELISA kits were used to test both experimental and field-collected sera in technical duplicates. The EHDV cELISA from Innovative Diagnostics (ID Vet, Grabels, France) and the BTV cELISA from IDEXX (Montpellier, France) were used according to manufacturer instructions. Field-collected sera, as well as positive and negative controls, were tested and read on a Synergy H4 Bio-Tek microtiter plate reader (Bio-Tek, Winooski, VT, USA) at 450 nm. Data collected were analyzed by Graph Pad Prism software for an ROC curve to determine positive and negative values.

### 2.6. Western Blots

Western blotting was used as a confirmatory test to further evaluate samples where FMIA and cELISA results did not agree. Whole virus (BTV or EHDV) was diluted to 4 Log_10_ plaque forming units (PFU)/mL in freshly prepared Laemmli Sample Buffer (Bio-Rad) and loaded into Mini-Protean TGX 4–20% gradient gels (Bio-Rad) with Precision Plus Protein WesternC standards (Bio-Rad) loaded between each sample lane for reference. Gels were run on a Bio-Rad electrophoresis unit (110 volts, 2 amps) for 1 h. Proteins were transferred from gels to nitrocellulose membranes using a Trans-Blot Turbo transfer system (Bio-Rad) with a Trans-Blot Turbo Mini 0.2 µm Nitrocellulose transfer pack (Bio-Rad) for 30 min (25 volts, 1 amp). Transferred membranes were stained for 3 min with 0.2% Ponceau S (Sigma Aldrich, St. Louis, MO, USA) to confirm protein transfer and rinsed briefly with Tris-buffered saline with 0.1% Tween-20 (TBST). Membranes were blocked overnight with protein-free blocking buffer (PBST) (G Biosciences, St. Louis, MO, USA) at 4 °C, cut into strips, each containing a lane of standards and a lane of virus sample, and placed into individual 15 mL tubes (Falcon Plastics) for subsequent antibody testing. The sera to be verified was added at 1:2000 in 10 mL of PBST and tubes were placed on a rotator (Labnet, Woodbridge, NJ, USA) to gently mix at room temperature for 1 h. Sera were removed, and strips were washed once with 10 mL of PBST with rotation for 15 min at room temperature. Wash was removed, 10 mL of rabbit anti-bovine IgG-horseradish peroxidase (HRP; Invitrogen) at 1:5000 in PBST was added, and tubes were rotated at room temperature for 30 min. The secondary antibody was removed, and strips were washed once as above with PBST. Next, strips were placed in flat staining dishes, covered with 5 mL of KPL True Blue Peroxidase Substrate (Sera Care, Milford, MA, USA), and placed in the dark to develop for 10 min. Color development was stopped with deionized water.

## 3. Results

### 3.1. Evaluation of Single-Analyte FMIA with Control Cattle Sera

**Figure 1:**
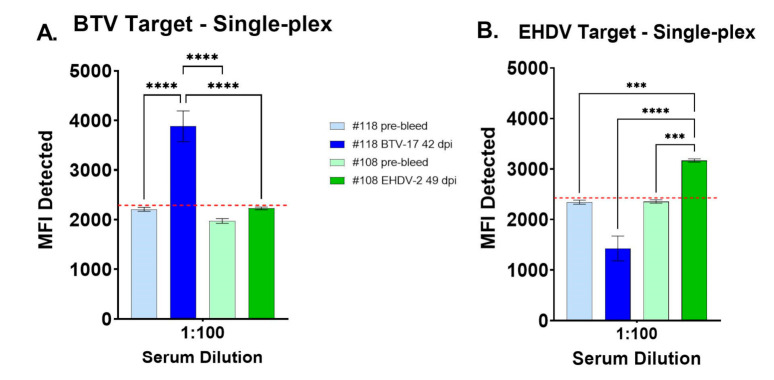
Mean fluorescence intensity (MFI) values of single-plex BTV and EHDV fluorescence microsphere immunoassays (FMIA) for pre-bleed and post-challenge cattle sera. (**A**) Single-plex BTV FMIA with sera from a BTV-17-inoculated cow (#118) at 42 days post-infection (dpi) compared to pre-bleed sera and sera from an EHDV-2-inoculated cow (#108) at 49 dpi. (**B**) Single-plex EHDV FMIA with sera from an EHDV-2-inoculated cow (#108) at 49 dpi compared to pre-bleed sera and sera from a BTV-17-inoculated cow (#118) at 42 dpi. One-way ANOVA with Dunnett’s multiple comparisons test and standard error; *** *p* < 0.001, **** *p* < 0.0001. Receiver operating characteristic (ROC) analysis followed by a Youden’s J Index was used to inform test cutoff value (red line) within the sera tested (BTV-target = 2288.46; EHDV-target = 2426.68).

Optimal whole-virus antigen protein concentrations were tested per manufacturer recommendations (Luminex Corporation). A concentration of 10 µg/mL of each viral antigen offered the best signal-to-noise ratio. Of the three commercially sourced bovine sera evaluated for use as a negative control, unfiltered normal cattle sera (Lampire Biological Laboratories) at 1:100 had MFI values most similar to pre-bleed cattle sera for both bead sets and was subsequently used in triplicate as a within-plate negative control for the BTV/EHDV duplex FMIA. Similarly, a dilution of 1:100 was determined to be optimal for the experimental BTV and EHDV positive control sera which were subsequently used to determine single-analyte specificity for virus conjugated microbeads. Results of the single-plex assays showed that for BTV-target microbeads, sera from a cow inoculated with BTV-17 at 42 dpi had significantly higher MFI than pre-bleed sera and sera from an EHDV-2-inoculated cow at 49 dpi (*p* < 0.0001) (Figure 1A). For EHDV-target microbeads, sera from the EHDV-2-inoculated cow at 49 dpi had significantly higher MFI than pre-bleed sera (*p* < 0.001) and sera from the BTV-17-inoculated cow at 42 dpi (*p* < 0.0001) (Figure 1B).

### 3.2. Evaluation of BTV/EHDV Duplex FMIA with Control Cattle Sera

**Figure 2:**
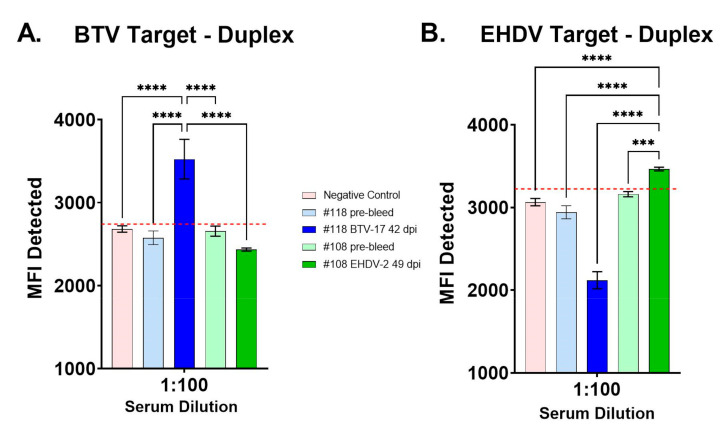
Mean fluorescence intensity (MFI) of the BTV/EHDV duplex FMIA for commercial negative control sera, pre-bleed sera, and post-challenge sera. (**A**) MFI results for the BTV-target microbeads with sera from a BTV-17-inoculated cow (#118) at 42 days post-infection (dpi) compared to commercial negative control sera, pre-bleed sera, and sera from an EHDV-2-inoculated cow (#108) at 49 dpi. (**B**) MFI results for the EHDV-target microbeads with sera from an EHDV-2-inoculated cow (#108) at 49 dpi compared to commercial negative control sera, pre-bleed sera, and sera from a BTV-17 -inoculated cow (#118) at 42 dpi. One-way ANOVA with Dunnett’s multiple comparisons test and standard error; *** *p* < 0.001, **** *p* < 0.0001. Receiver operating characteristic (ROC) analysis followed by a Youden’s J Index was used to inform test cutoff value (red line) within the sera tested (BTV-target = 2740.06; EHDV-target = 3224.97).

Negative and positive controls were used to evaluate the duplex FMIA for simultaneous detection of BTV and EHDV antibodies with both conjugated bead sets in the same well (Figure 2). For the BTV-target microbeads, sera from a BTV-17-inoculated cow at 42 dpi was significantly higher than negative control sera (Lampire), pre-bleed sera, and sera from an EHDV-2-inoculated cow at 49 dpi (*p* < 0.0001). For the EHDV-target microbeads, sera from an EHDV-2-inoculated cow at 49 dpi was significantly higher than negative control sera and pre-bleed sera (*p* < 0.001 or *p* < 0.0001), and sera from a BTV-17-inoculated cow at 42 dpi (*p* < 0.0001). No cross-reactivity was seen with EHDV-2 antisera for the BTV-target microbead, nor with BTV-17 antisera for the EHDV-target microbead.

**Figure 3:**
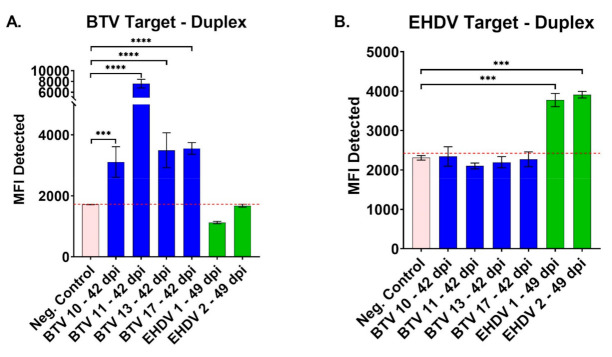
Mean fluorescence intensity (MFI) values of the BTV/EHDV duplex FMIA for simultaneous, differential detection of antibody to multiple BTV and EHDV serotypes. (**A**) MFI values for the BTV-target microbead with sera from cattle experimentally infected with BTV-10, -11, -13, -17 or EHDV-1 or -2, compared to commercial negative control sera. (**B**) MFI values for the same negative control and experimental sera against the EHDV-target microbead. Two-way ANOVA with Dunnett’s multiple comparisons test and standard error; *** *p* < 0.001, **** *p* < 0.0001. Receiver operating characteristic (ROC) analysis followed by a Youden’s J Index was used to inform test cutoff value (red line) within the sera tested (BTV-target = 1724.43; EHDV-target = 2423.51).

In addition to the initial evaluation with BTV-17 and EHDV-2 antisera, the duplex FMIA was further evaluated with available experimental sera for its ability to detect antibodies to other domestic BTV and EHDV serotypes: BTV-10, -11, and -13, and EHDV-1 (Figure 3). Previous plaque reduction neutralization tests for 80% reduction (PRNT_80_) with these experimental sera showed homologous PRNT_80_ antibody titers of 1:320 for all four BTV antisera at 42 dpi and antibody titers of 1:640 for the two EHDV sera. The MFI for the BTV-target microbeads showed sera from all four BTV-inoculated cattle (42 dpi) were significantly higher than negative control sera (*p* < 0.001 or *p* < 0.0001). No cross-reactivity was seen with EHDV-1 and -2 antisera for BTV-target microbeads. The MFI for the EHDV-target microbeads showed sera from both EHDV-1 and EHDV-2 infected cattle (49 dpi) were significantly higher than negative control sera (*p* < 0.001). No cross-reactivity was seen with BTV-10, -11, -13, or -17 antisera for EHDV-target microbeads.

### 3.3. Validation of Duplex FMIA with Field-Collected Cattle Sera

**Figure 4:**
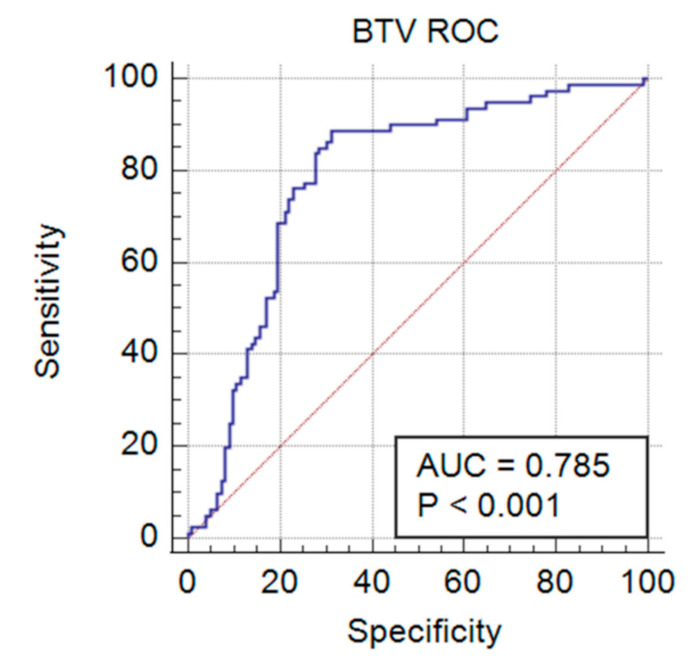
Receiver operating characteristic (ROC) curve showing the performance of the BTV analyte in the BTV/EHDV duplex FMIA at all classification thresholds. The area under the curve (AUC) of 0.785 (*p* < 0.001) gave an acceptable relationship [51] between the ability of the FMIA to detect BTV antibody-positive samples when compared to the BTV cELISA.

**Figure 5:**
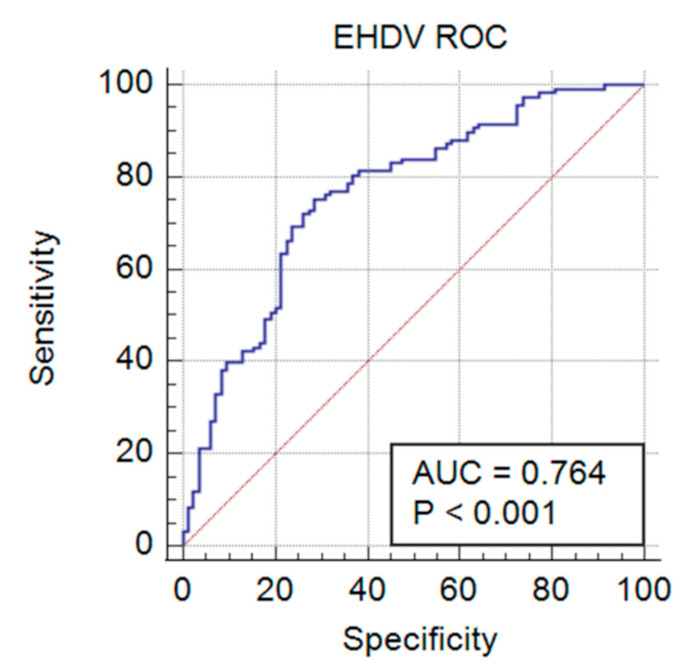
Receiver operating characteristic (ROC) curve showing the performance of the EHDV analyte in the BTV/EHDV duplex FMIA at all classification thresholds. The area under the curve (AUC) of 0.764 (*p* < 0.001) gave an acceptable relationship [51] between the ability of the FMIA to detect EHDV antibody-positive samples when compared to the EHDV cELISA.

Once optimized with the negative and positive control cattle sera, 202 field-collected cattle sera were tested to validate the BTV/EHDV duplex FMIA. Outbreak sera with known BTV/EHDV cELISA serological status (*n* = 89), and surveillance sera with known BTV/EHDV AGID serological status (*n* = 113), were tested in technical triplicates. The MFI values from each target microbead set were converted to sample value/positive value (S/P) ratios as above, to compare between plates and known previous serological data, with statistical analysis via ROC and Youden’s J Index to determine sensitivity, specificity, and to rate the performance of the assay. For the duplex FMIA detection of BTV antibody, an ROC area under the curve (AUC) was 0.785 (*p* < 0.001) with a 95% confidence interval (CI) of 0.722 to 0.840 (Figure 4). Thus, the aggregate measure of performance for the duplex FMIA for detecting BTV antibody across all possible classification thresholds was 78.5%. The Youden’s J Index of 0.5760 from this ROC directed the S/P criterion of >−0.0167 with 88.75% sensitivity (95% CI = 79.7–94.7) and 68.85% specificity (95% CI = 59.8–76.9) (Table 1). For detection of EHDV antibody, the ROC AUC was 0.764 (*p* < 0.001), with a 95% CI of 0.699 to 0.821 (Figure 5). Thus, the aggregate measure of performance for the duplex FMIA for detecting EHDV antibody across all possible classification thresholds was 76.4%. The Youden’s J Index of 0.4685 from this ROC directed an S/P criterion value > 0.0623 with a sensitivity of 75.42% (95% CI = 66.6–82.9) and a specificity of 71.43% (95% CI = 60.5–80.8) (Table 2).

**Figure 6:**
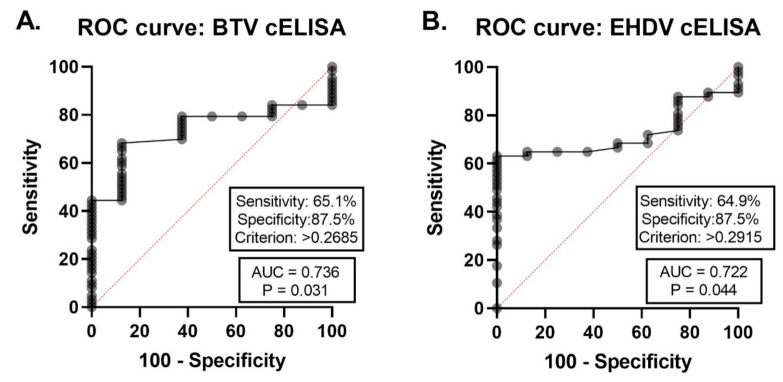
Receiver operating characteristic (ROC) curve showing the performance of the commercial BTV and EHDV cELISA kits for 89 field-collected cattle serum samples. (**A**) The area under the curve (AUC) reported an acceptable relationship [51] of 0.736 (*p* = 0.031) between the BTV cELISA kit (IDEXX) and its controls. (**B**) The AUC reported an acceptable relationship of 0.722 (*p* = 0.044) between the EHDV cELISA (ID Vet) and its controls. ROC curves were based on absorbance of samples compared to kit controls. Sensitivity, specificity, and criterion results as indicated.

The 89 field-collected sera of unknown serological status were also tested by BTV cELISA (IDEXX) and EHDV cELISA (IDVet). These results were then examined for an ROC curve to determine cutoffs for later comparison to the FMIA data (Figure 6). Of the 89 samples, 14 were had antibody to both viruses, 1 had BTV antibody only, 36 had EHDV antibody only, and 38 were double negatives. The AUC for the BTV cELISA reported as 0.736 (*p* = 0.031, 95% CI = 0.6104 to 0.8618). Thus, the aggregate measure of performance for the BTV cELISA for detecting BTV antibodies across all possible classification thresholds was 73.6%. The Youden’s J Index from this ROC directed an S/P criterion of >0.2685 at 65.1% sensitivity (95% CI = 52.75% to 75.67%) and a specificity of 87.50% (95% CI = 52.91% to 99.36%). The EHDV cELISA reported an AUC of 0.722 (*p* = 0.044, 95% CI = 0.5983 to 0.8446). Thus, the aggregate measure of performance for the EHDV cELISA for detecting EHDV antibodies across all possible classification thresholds was 72.2%. The Youden’s J Index from this ROC directed an S/P criterion of >0.2915 at 64.91% sensitivity (95% CI = 51.94% to 76.00%) and a specificity of 87.50% (95% CI = 52.91% to 99.36%).

Of the 202 field-collected sera, the duplex FMIA detected 96 samples with antibody to both viruses, 10 with antibody to BTV only, 25 with antibody to EHDV only, and 71 double negatives. Results of the BTV-target in the duplex FMIA disagreed with the BTV cELISA 11 times (5%) and agreed with the Western blot results for those discrepancies 63% of the time. Results of the EHDV-target in the duplex FMIA disagreed with the EHDV cELISA 14 times (7%) and agreed with the Western blot results for those discrepancies 38% of the time. In these cases, the Western blot results were used for true positives to determine the input for ROC and Youden’s J Index analyses.

## 4. Discussion

The hemorrhagic orbiviruses, BTV and EHDV, cause significant economic losses to the cattle industry primarily due to animal movement and trade restrictions. For international trade purposes, cattle must be certified as testing serogroup-negative for both viruses [49]. The most commonly used serogroup-specific BTV and EHDV serological tests, for both research and veterinary diagnostic laboratories, are the AGID and cELISA. The AGID is simple to perform, inexpensive, and the antigen is easy to produce. However, due to high rates of cross-reactivity between BTV and EHDV with AGID [46,52], the OIE states that this assay is not appropriate for determining the negative serological status of an individual animal prior to movement, nor for confirmation of clinical cases [49]. Commercially available cELISA assays are sensitive, specific, high throughput, and reliable in detecting serogroup antibody without the cross-reactivity problems of the AGID. However, kits are expensive, and samples must be tested individually with an ELISA specific for each virus requiring more sera, time, effort, and cost to establish an animal as having a double-negative serological status. No commercial cELISA kits allow differential testing for both viruses simultaneously in a single serum sample. Additionally, for U.S. diagnostic laboratories, obtaining some kits requires import permits and expensive international shipping.

In the past decade, FMIA technology to detect antibody to a single animal disease pathogen [53,54,55,56,57,58], or to multiple pathogens [59,60] in a single serum sample has been successful. The initial investment is higher than ELISA platforms, as the MAGPIX^®^ instrument is more expensive than an optical density ELISA plate reader; however, FMIA technology provides simultaneous detection of multiple analytes (up to 500) in a single sample, with high sensitivity and specificity, and with significant time and cost savings per sample, compared to single-plex ELISAs.

Initial testing of the BTV/EHDV duplex FMIA reported here shows promise as a new platform for detecting and differentiating the orbivirus serological status of cattle for surveillance and trade regulation. While subsequent plaque neutralizations would still be required to determine the specific serotype to which cattle were exposed in order to inform vaccination efforts, regulatory/trade restrictions are based solely on serogroup status, regardless of serotype. Overall, the assay’s performance with experimental and field sera were consistent, sensitive, and specific in simultaneously detecting and differentiating BTV and EHDV antibodies in a single sample. Compared to the IDEXX commercial BTV cELISA, the duplex FMIA had higher performance in detecting BTV antibody (78.5% vs. 73.6%), with higher sensitivity (88.75% vs. 65.1%) but lower specificity (68.85% vs. 87.5%). Compared to the ID Vet commercial EHDV cELISA, the duplex FMIA had higher performance in detecting EHDV antibody (76.4% vs. 72.2%), again with higher sensitivity (75.42% vs. 64.91%) but lower specificity (71.43% vs. 87.50%). While both are central to diagnostic reliability, sensitivity and specificity have different purposes. The most successful diagnostic assays find an optimal balance so that sensitivity is not sacrificed for specificity, and test results can be trusted to inform appropriate decision making [61].

For the implementation of FMIA platforms in research and diagnostic laboratories, it should be recognized that FMIA data do not fit a normal distribution. Therefore, ROC analyses are used to allow for a statistical, non-parametric determination of the assay thresholds for true positives and negatives. The use of a commercially available negative control reagent that tests accurately across all plates, such as that used here, is advantageous for comparing analyses between plates and laboratories. Additionally, some ELISA kit negative controls test far below normal bovine sera in terms of signal-to-noise ratios. This can create an artificially low negative cut-off value, resulting false positives. The negative control sera used here, was representative of uninfected field-collected cattle sera. Future optimization could utilize internal control microbeads to further increase accuracy and precision of the test, while also monitoring instrument fluctuations [62]. A limitation of the FMIA, as with cELISA and AGID assays, was a tendency for false results from archived sera with evidence of fungal growth [63].

Effective prevention, response, trade regulation, and control of BTV and EHDV in cattle requires a well-developed variety of tools and approaches that can be employed for surveillance and rapid detection. In this study, we developed a duplex fluorescent microsphere serological assay for BTV and EHDV and evaluated it with field-collected cattle sera. The results demonstrate comparable overall performance, compared to the cELISA platforms, with higher sensitivity and the additional ability to simultaneously detect and differentiate the serogroup antibodies in a single, minimal sample volume. Benefits of the BTV/EHDV duplex FMIA platform, over current diagnostic methods of cELISA and AGID, include the ability for simultaneous, rapid, and accurate identification of livestock herd immune status for two orbiviruses in a high-throughput capacity. This reduces the time required for trade regulation testing, for implementing possible quarantine or containment efforts, and for determining whether orbiviruses circulating in a cattle herd are BTV or EHDV and therefore a risk, or not, to nearby sheep flocks. Furthermore, the BTV/EHDV duplex FMIA could be expanded, and its cost-effectiveness enhanced, by adding targets for other cattle pathogens with similar clinical disease. One potential example of this could be a bovine nasal discharge diagnostic panel to include bovine respiratory syncytial virus, bovine viral diarrhea virus, infectious bovine rhinotracheitis virus, and parainfluenza type 3 virus. Another example could be a bovine abortion diagnostic panel to include *Neospora caninum*, *Brucella abortus*, *Leptopira*, bovine viral diarrhea virus, infectious bovine rhinotracheitis virus, Schmallenberg virus, and Rift Valley fever virus.

## Figures and Tables

**Table 1:** Sample value/positive value (S/P) criterion of positivity (>−0.0167 *) for the BTV analyte in the BTV/EHDV duplex FMIA. MedCalc was used to determine sensitivity and specificity at a 95% confidence interval (CI).

Criterion	Sensitivity	95% CI	Specificity	95% CI	+LR ^1^	−LR ^2^
>−0.1061	90.00	81.2–95.6	55.74	46.5–64.7	2.03	0.18
>−0.1013	88.75	79.7–94.7	55.74	46.5–64.7	2.01	0.20
>−0.0167 *	88.75	79.7–94.7	68.85	59.8–76.9	2.85	0.16
>−0.0003	86.25	76.7–92.9	68.85	59.8–76.9	2.77	0.20
>−0.0004	86.25	76.7–92.9	69.67	60.7–77.7	2.84	0.20

^1^ Positive likelihood ratio. ^2^ Negative likelihood ratio.

**Table 2:** Sample value/positive value (S/P) criterion of positivity (>0.0623 *) for the EHDV analyte in the BTV/EHDV duplex FMIA. MedCalc was used to determine sensitivity and specificity at a 95% confidence interval (CI).

Criterion	Sensitivity	95% CI	Specificity	95% CI	+LR ^1^	−LR ^2^
>0.0401	76.27	67.6–83.6	69.05	58.0–78.7	2.46	0.34
>0.0437	75.42	66.6–82.9	69.05	58.0–78.7	2.44	0.36
>0.0623 *	75.42	66.6–82.9	71.43	60.5–80.8	2.64	0.34
>0.089	72.88	63.9–80.7	71.43	60.5–80.8	2.55	0.38
>0.1019	72.88	63.9–80.7	72.62	61.8–81.8	2.66	0.37

^1^ Positive likelihood ratio. ^2^ Negative likelihood ratio.

## Data Availability

The data presented in this study are available on request from the corresponding author.
